# Fake spawns and floating particles: a rebuttal of Karkarey et al. “Alternative reproductive tactics and inverse size-assortment in a high-density fish spawning aggregation”

**DOI:** 10.1186/s12898-018-0206-8

**Published:** 2018-11-27

**Authors:** Brad E. Erisman, João P. Barreiros, Kevin L. Rhodes, Robert R. Warner

**Affiliations:** 1 Marine Science Institute, The University of Texas at Austin, 750 Channel View Drive, Port Aransas, TX 78373-5015 USA; 2 CE3C-Centre for Ecology, Evolution and Environmental Changes/Azorean Biodiversity Group and Faculty of Agrarian and Environmental Sciences, University of the Azores, 9700-042 Angra do Heroísmo, Portugal; 3 Marine Laboratory, University of Guam, UoG Station, Mangilao, Guam 96923 USA; 4 Department of Ecology, Evolution, and Marine Biology, University of California Santa Barbara, Santa Barbara, CA 93016 USA

**Keywords:** Spawning aggregation, High mating density, Alternative reproductive tactics, Shoal and pair courtship tactics, Inverse size-assortment, Squaretail coralgrouper

## Abstract

Courtship and spawning behaviors of coral reef fishes are very complex, and sufficient sampling effort and proper methods are required to draw informed conclusions on their mating systems that are grounded in contemporary theories of mate choice and sexual selection. We reviewed the recent study by Karkarey et al. (BMC Ecol 17:10, 2017) on the spawning behavior of Squaretail coralgrouper (*Plectropomus areolatus*) from India and found no evidence to support their findings of alternative reproductive tactics, unique school-spawning involving a single male with multiple females, or inverse size-assortment. The study lacks scientific credibility due to a lack of rigor in the methodology used, misinterpretation of observed behaviors, misinterpretation of the literature, and insufficient data. Their approach led the authors to produce spurious results and profound, invalid conclusions that violate the most basic assumptions of mate choice and sexual selection theory as applied to mating systems in marine fishes.

## Main text

In a recent issue in this journal, Karkarey et al. [1] conducted an observational study of the mating system of the Squaretail coralgrouper (*Plectropomus areolatus*) at a “pristine” site off Bitra, a remote atoll in the northern Lakshadweep archipelago off India. As part of their principal findings, Karkarey et al. [1] described an extraordinary mating behavior referred to as a “unique school-spawning tactic” in which multiple females group-spawned with a single male. This mating style has never been reported in any broadcast spawning species of marine fish, and as such, it would be a unique and important discovery. Similarly, they also reported that *P. areolatus* at Bitra showed a habitat-specific, inverse size-assortment in relationship to courtship in which “large males courted small females on the reef slope while small males courted equal-sized or larger females on the shelf.” Both of these reported mating behaviors would appear to violate the most basic assumptions of mate choice and sexual selection theory as applied to marine fishes, and thus the study demanded further scrutiny.

After careful consideration, we report here that the results of Karkarey et al. [1] are unsupportable and their conclusions are invalid, as both were based on false interpretations of behavioral observations, a lack of rigor in the methods chosen, and a lack of data to support their conclusions. In discussing their findings, the authors failed to provide any explanation on the proximate or ultimate causes of such unique behaviors that were grounded in or supported by empirical evidence from contemporary theories on sexual selection, mate choice, and mating systems in marine fishes. The legitimacy of the study was further undermined by the exclusion, misinterpretation, and improper citation of previous studies on the mating behavior of *P. areolatus* and other marine fishes and seminal literature on mating systems and sexual selection. Based upon the serious issues contained in the study, which we grouped into five categories described below, we concluded that the study by Karkarey et al. [1] lacks scientific merit and should be retracted from the literature.

### (1) False observations of spawning events involving a single male with multiple females

Karkarey et al. [1] claim they observed two instances (once each in 2013 and 2014) of a unique and novel “school-courtship culminating in gamete release”, each involving a single male and 4 to 5 female *P. areolatus* from within a larger school of females. To support their claim, they provide a photograph in the manuscript (Fig. 2d in [1]) taken from a video clip of the first supposed spawning event observed in 2013, which is also included in the publication as supplementary information (Additional file 2 in [1]). The authors contend that the photograph and the video clip reveal a “school-spawning tactic” that “involved an upward spawning rush within the school in the water column commonly seen in mass-spawning fish”. They also explain that females involved in the observed spawning event could be “clearly identified based on their distended bellies” and “simultaneously released gametes as a cohesive unit”.

Each of us has observed the video file numerous times, both in real time and in slow motion, and we can find no plausible evidence of spawning or of any of the behaviors described by Karkarey et al. [1]. In direct contrast to the article’s description of spawning in the video, the groupers swim rapidly downward toward the reef rather than upward, with individuals colliding briefly in a very disorganized manner before dispersing. The event ends with the lead individual (small, with dark color phase typical of females; see page 44 in [2]) involved in the event swimming rapidly downward and into the reef as it is pursued by a larger individual exhibiting the camouflage color phase (which can be either a male or a female [2]). Most of the individuals involved in the incident appear to exhibit the dark color phase typical of schooling females [2]. The “spawning” event occurs far in the distance, with no individuals with distended bellies apparent, suggesting these individuals are not females ready to spawn.

**Fig. 1:**
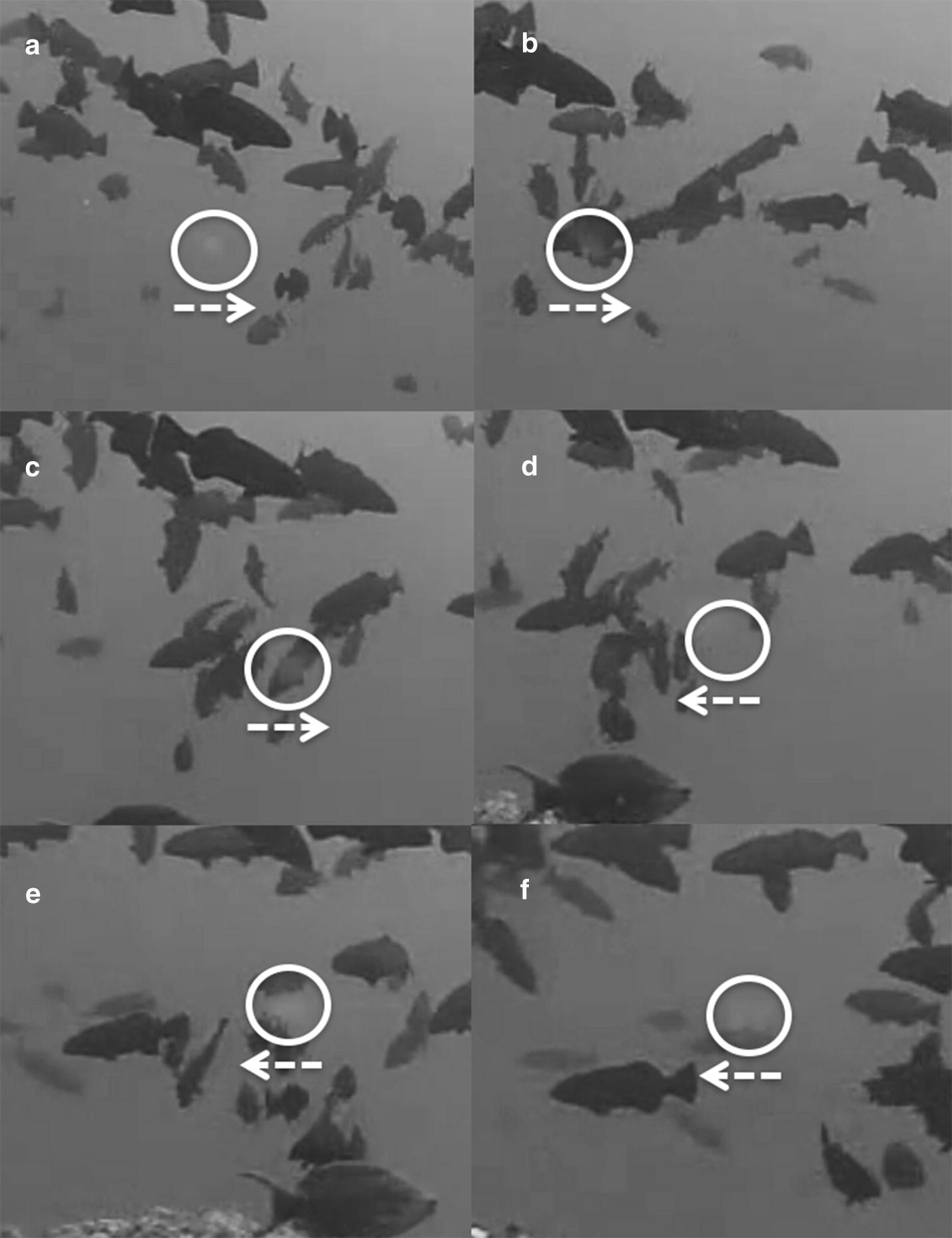
Sequence of still frames (**a**–**f**) extracted from Additional file 2 of Karkarey et al. [1] showing the location (white circle) and the general direction of movement (white arrow) of the particle misrepresented as a gamete cloud. Please note that Fig. 2d in Karkarey et al. [1] would occur between the still frames in (**c**) and (**d**)

**Fig. 2:**
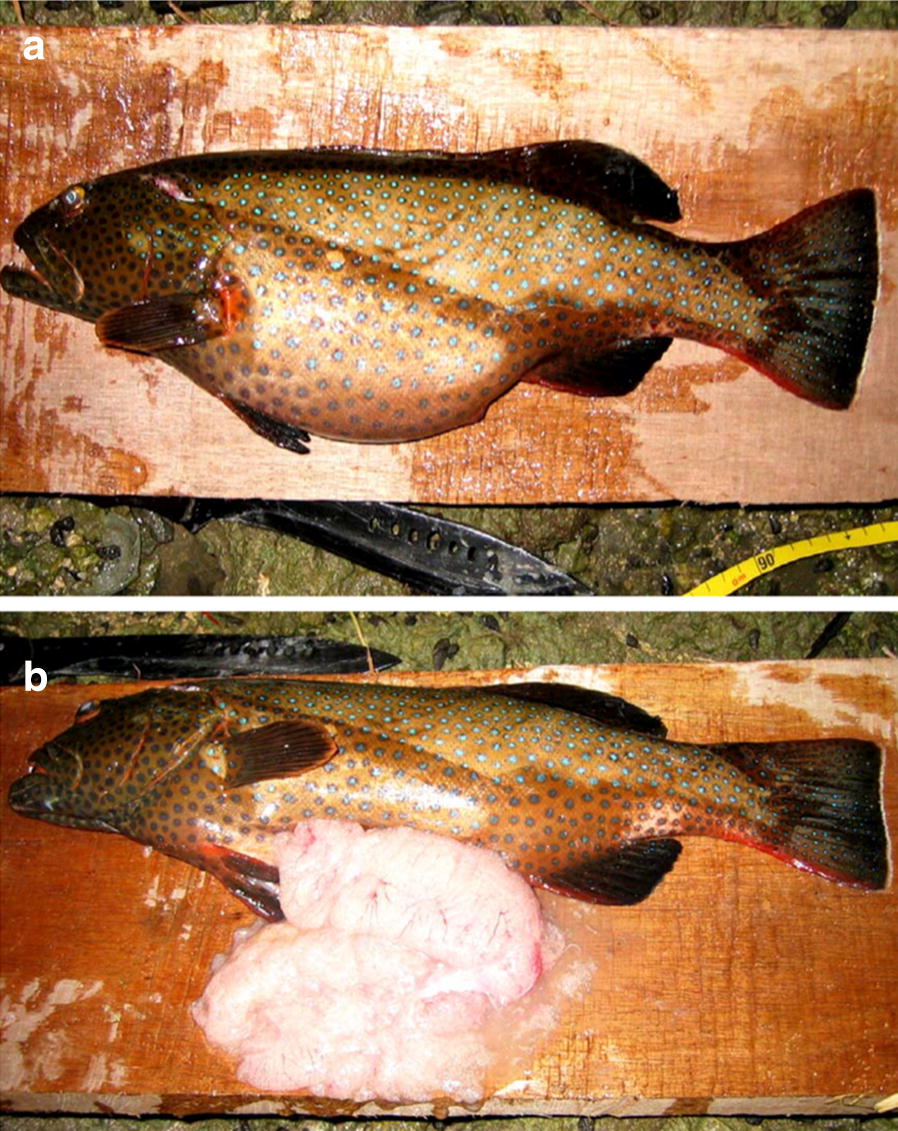
Photographs of a gravid female *P. areolatus* with: **a** a highly distended abdomen indicating that spawning is imminent; and with **b** gonads dissected to verify that the process of ovulation (as evidenced by the clear oocytes leaking onto the table) had begun at the time of collection (Photo provided by Richard Hamilton)

Further inspection of the proposed “gamete cloud” (circled in Fig. 2d in [1]) served as the most conclusive evidence that the event shown in the video and the photograph was not associated with spawning. Contrary to descriptions provided by Karkarey et al. [1], no simultaneous release of eggs by females can be seen at any point in the video. Notably, ovulated grouper eggs that are shed into the water column during broadcast spawning events are almost completely translucent and difficult to see from any distance (BEE, KLR, JPB, RRW pers. obs.). Upon reviewing the video in slow motion, we confirmed the object circled in Fig. 2d of Karkarey et al. [1] is not a gamete cloud at all but rather a small, white particle drifting in the water column very close to the camera lens. The particle appears clearly in the clip at 0:14 s, during the slow motion footage, where it is located in open water just below the center of the aggregation. It then moves from left to right and passes rapidly across the camera lens and in directly in front of the group of fish as they come together (0:15 s), giving the false appearance of a possible, faint, small, gamete cloud when viewed casually at normal speed. Immediately after, it becomes clear that the ‘gamete cloud’ is a particle, as the object changes directions completely and moves rapidly from right to left in a different direction, on a different plane, and at a much faster rate than any of the fish associated with the event. The particle exits the screen at 0:18 s of the video clip, while passing in front of other fish that are viewable on the screen. We provide still frames of the particle and its movement in Fig. 1 and encourage readers to carefully compare this sequence with Fig. 2d and the supplementary video provided by Karkarey et al. [1] to draw their own conclusions.

### (2) No empirical evidence of single male–multiple female spawning in *P. areolatus*, groupers, or any other coral reef fish

Karkarey et al. [1] misrepresents the novelty of female *P. areolatus* schools. As early as 1999, Johannes et al. [2] reported that the “schools of female *P. areolatus* swimming to, from or within spawning aggregations seem to be the only example of single-sex schooling behavior we know of among groupers within this genus”. Johannes et al. [2] also described in great detail that when roving schools of females passed over males occupying territories on the reef, the males would “break quickly into the school [of females], pushing sideways vigorously with their bodies against females, apparently trying to separate them from the school… In apparent response to these efforts, individual females would leave the school and move to the bottom.” Johannes et al. [2] proposed several plausible explanations for the formation of female schools, including anti-predator measures and protection of harassment and mobbing by males. Karkarey et al. [1] cite the work done by Johannes et al. [2] but failed to discuss these conclusions in their manuscript.

Karkarey et al. [1] also observed males making forays into the schools (see Fig. 2c in [1]), which they concluded represented a courtship act between the acting male and multiple females. The authors also claimed these observations “reveal an additional school-associated courtship tactic, distinct from earlier reports in the literature for this species”. This explanation differs greatly from those by Johannes et al. [2] and the individual authors listed therein, who based their explanation on decades of combined experience of grouper mating from numerous spawning aggregations in multiple locations globally, in addition to the several years of continuous surveys and observations of *P. areolatus* at the site of their reported research. Descriptions by Johannes et al. [2] of interactions between territorial males and roving female schools closely resemble the sequence of behaviors that can be seen in the video clip provided by Karkarey et al. [1] and served as further evidence to invalidate claims of a unique school-spawning tactic involving a single male and multiple females. Specifically, one can observe several instances of putative males darting up from the reef and into the school, including the false spawning event that ends with the lead fish (exhibiting the typical color phase of schooling females [2]) swimming down from the school and into the reef while being pursued by a putative male (based on bicolor phase; see [2]).

The sequence of behaviors in the video provided by Karkarey et al. [1] bears no resemblance to verified group courtship and spawning events described in other species of groupers. In all species studied to date, the courtship and pre-spawning period involve several males that chase, harass, mob, and eventually surround a single, gravid female in a very prescribed, cylindrical orientation [3–5]. These behaviors eventually lead to a coordinated spawning rush of the group upwards towards the surface and ending in the simultaneous release of large volumes of gametes that are visibly evident by a large cloud of milt. Similar behaviors have also been documented in numerous species of wrasses, parrotfishes, snappers, surgeonfishes, and other reef fishes with group-spawning mating systems [6–10].

Previous studies on *P. areolatus* provide evidence that the species does demonstrate two types of ARTs much like many other coral reef fishes with external fertilization (reviewed by [11]) but not those described by Karkarey et al. [1]. Johannes et al. [2] described one instance of unverified pair spawning, and Pet et al. [12] described four pair spawning events between a territorial male and an individual female, indicating that male *P. areolatus* do engage in the typical, large male, mate monopolization tactic of other groupers and coral reef fishes. Likewise, descriptions of courtship behavior by Johannes et al. [2] indicate that *P. areolatus* is likely to spawn in single female–multiple male groups at high densities with intense sperm competition. At two sites in Palau, during the peak aggregation days when fish abundance and spawning activity (deduced from histological analysis of collected fish) were highest, they reported that sex ratios were highly biased towards males at the core of the aggregation. The authors of the study reported that “courting behavior …seemed to reflect this shortage of females. Females were often harassed (nudged, chased, collided with) by several males simultaneously and often fled from them.” In the areas where fish densities were highest, up to 40 males were observed engaging in this behavior. Johannes et al. [2] noted that the intensity and duration of male–male interactions and color changes were higher during these periods and at these locations, and that chasing of females by individual and groups of males were more common at these locations than elsewhere within the aggregations and at other aggregation sites where sex ratios were less skewed towards males. Collectively, all of the descriptions of multiple male–single female courtship behaviors described by Johannes et al. [2] match those documented in other species of groupers with group-spawning mating systems [4, 5, 13].

### (3) No theoretical support for single male–multiple female spawning in broadcast spawning fishes

From a broader perspective, the conclusions of Karkarey et al. [1] that *P. areolatus* at Bitra exhibit a unique spawning tactic involving individual males simultaneously mating with multiple females holds serious implications for sexual selection theory and mating systems of groupers and other marine fishes. Anisogamy (different-sized male and female gametes) generally leads to situations in which male gametes and individual males are in competition with each other to access and fertilize the eggs produced by females [14–16]. This competition can take many forms, including sperm competition in multi-male group spawns with individual females when sperm and eggs are released externally [11, 17, 18]. Moreover, reviews of mating behaviors in groupers have revealed remarkable support for these prevailing theories of sexual selection and sperm competition: In accordance with these predictions, groupers that form large spawning aggregations with a high density of individuals competing for matings with females all engage in group-spawning events involving multiple males and a gravid female [13, 19].

While the general conclusion by Karkarey et al. [1] that “school courtship” can lead to “school spawning” in high density aggregations of reef fishes is supportable, their description of mating tactics in high-density populations of marine fishes is inaccurate. The authors claim that under high-density situations, if a few individuals are able to monopolize matings, then others will have little success. In contrast, studies of mating behavior in broadcast-spawning reef fishes have unilaterally demonstrated that mate monopolization is *negatively* correlated with population density [20–22]. That is, the success rate of territorial males decreases as the population density of smaller males increases. Under high densities, territoriality and pair spawning are replaced by group-spawning tactics and high investment in sperm production due to increased sperm competition [7, 21, 22], a scenario that has been demonstrated in both empirical studies of individual fish populations and phylogenetic studies of mating system evolution in fishes [19]. Therefore, to say that high population density “requires individuals to adopt innovative mating strategies” [1] is inaccurate and ignores the substantial literature on the influence of density on mating systems in reef fishes.

Contrary to prevailing theories supported by extensive empirical evidence and numerous case studies of coral reef fishes, Karkarey et al. [1] appear to invoke some type of “egg competition” where multiple females are competing to have their eggs fertilized by a single male. How is this justified or explained within the context of reproductive fitness? Why would multiple females choose to risk their eggs on a single male’s sperm release when numerous other males are present? If Karkarey et al. [1] are going to put forth a theory that violates the most basic assumptions of mate choice and sexual selection theory as applied to marine fishes, at the very least, a plausible explanation posed within the proper theoretical context needs to be provided—we are certainly unable to do so. Instead, they provide no theoretical explanation or any empirical evidence to support their findings of a novel school-spawning tactic in *P. areolatus*, rendering the principal finding of their study and all associated conclusions regarding the existence, costs, and benefits of alternative reproductive tactics in the species as unsupportable and lacking scientific merit.

### (4) Insufficient evidence that observations were made during the actual spawning period

Karkarey et al. [1] provided no details on the exact dates or time of day of their observations, but from the evidence presented, their behavioral surveys were likely carried out prior to the actual spawning period. Extensive work by Johannes et al. [2] in Palau and Rhodes et al. [23–25] in Pohnpei showed that roving schools of female *P. areolatus* are most commonly observed on the reef several days prior to spawning. During this time period, oocytes have not progressed to the point of hydration [2, 23, 25]. Female schools represent the first entry of females into the aggregation, with males having established territories over a few days prior [23]. Female schools tend to dissipate rather rapidly thereafter, with individual females dispersing throughout the aggregation area, where they are engaged by individual males within the males’ established territories [2, 23–25]. Males show aggression toward females during these initial engagement periods, with chasing quite common. As the time of spawning nears, females mingle among male territories and courtship ensues. Courtship results in eventual pair-spawning, with spawning during the aggregation period occurring over 2–3 days, as indicated by reductions in density following peak abundance periods [12, 25].

During the day(s) of actual spawning in *P. areolatus*, the abdomens of females are remarkably distended with hydrated eggs (Fig. 2). The process of ovulation that occurs just before spawning causes females to swim oddly and sluggishly, and makes it easy to identify that spawning is imminent (BEE, KLR, RRW pers. obs.). Strikingly, no highly gravid (hydrated or ovulated) females could be seen among the “roving school of females” in either the photos or the videos provided in Karkarey et al. [1]. Likewise, the photo provided by Karkarey et al. [1] as an example of paired courtship (Fig. 2c in [1]) provides no definitive evidence that either individual is a gravid female, suggesting that the interaction does not reflect courtship.

### (5) No evidence of “inverse size-assortment” due to invalid methods used to estimate courtship rates

Karkarey et al. [1] contend they made numerous observations to describe courtship behaviors and to draw conclusions about the benefits of various types of courtship observed (e.g. association rates of small vs. large males with females; paired vs. group spawning, etc.). Based on their results, they posited that *P. areolatus* showed “a habitat-specific inverse size-assortment”, in which “large males courted small females on the slope, while small males courted equal-sized or larger females on the shelf”. However, their methods and results suffer from a serious flaw: the authors cannot claim the behaviors they observed were actually courtship or representative of inverse-size assortative mating unless they are at least sometimes followed by a spawning event. The authors themselves state they never once observed a spawning event between a male–female pair of fish. The only evidence they presented to justify their findings were that these behaviors were observed at spawning aggregations of *P. areolatus* in previous studies [2, 12].

While it is unclear whether courtship was measured at all, it is never appropriate to measure courtship (i.e. the number of females being courted) as a “benefit” accruing to males, because it is unknown whether these behaviors led to successful spawning or whether these individuals remained in the observation arena until spawning commenced. The authors did not observe mating in either large or small individuals, so it is also unreasonable to draw conclusions about inverse size-assortative mating. Additionally, local sex ratio (also potentially incorrectly measured, see below) contaminates the courtship rate measurements, because the “benefit” is multiplied by the number of nearby females; this leads to an estimate of higher “benefit” for males on the shelf, even though time spent in “courtship” was claimed as the same in both habitats. This is the approach taken by Karkarey et al. [1], as shown in Fig. 3 of their study. The results presented in the figure show no difference in “courtship” rates among any combination of size or habitat type except for those by large males with schools of small females on the slope. This difference is an artifact of the authors counting all the females in the vicinity of the males (i.e. on a per female basis) as being simultaneously courted by that individual male rather than counting the interaction as a single event.

It is implausible to make sound inferences about mating rates, potential mating opportunities, or costs associated with intra-sexual selection when none of the measurements used to generate them were based on verified reproductive activity. The observations by Karkarey et al. [1] of male–female encounters could represent mating opportunities, but only if we assume that the encounters took place during the spawning period. Unfortunately, the evidence presented above suggests this is not the case, and the authors can only make claims about association rates between individuals, possibly between males and females, but without reference to any measured component of fitness. They cannot make any statements about mating opportunities, because there is no understanding of how the spatial dynamics of small or large individuals, territorial males, or female schools change as the timing of spawning approaches. Since no spawning was observed in either large or small males, claims related to alternative reproductive tactics (ARTs), inverse size-assortative mating, mating success, mating rates, and “maximizing mate quantity” represent nothing more than unsubstantiated speculations. In fact, even conclusions drawn by Karkarey et al. [1] about association rates are questionable, since all their data were gathered from observations made during only two survey days over 2 years (see Table 3 in [1]).

## Conclusions

Conducting comprehensive quantitative analyses of fish mating behavior, courtship and mating rates, and related factors requires careful, appropriate design and analyses. These practices can result in novel findings that propel our interest and understanding in these and other organisms; however, rarely do these findings contradict established theory. In these instances, clear, irrefutable evidence is required that is supported by rigorous methodology, observations, and analyses. While Karkarey et al. [1] should be recognized for attempting to advance our understanding of the reproductive dynamics of Squaretail coralgrouper and apply their findings towards conservation efforts, their published findings are unsupportable based on the evidence presented. We therefore reject the authors’ claims of “unique school spawning” involving a single male and multiple females given the lack of any evidence of gamete release during a period when reproduction was unlikely to occur and suggest the evidence provided is reflective of events described nearly two decades ago by Johannes et al. [2]. We also refute their conclusions regarding inverse size-assortative mating and courtship rates due to the use of improper methodologies and insufficient sampling effort that produced results with no verification of reproductive activity. Despite the spurious results and invalid conclusions of the study, we encourage the authors of Karkarey et al. [1] to broaden and strengthen their future work in order to provide a better, yet sound, understanding of the reproductive dynamics of this vulnerable species.
